# Dataset of seven tropical flower species from Bangladesh: A resource for classification and ecological studies

**DOI:** 10.1016/j.dib.2025.111374

**Published:** 2025-02-07

**Authors:** Riazul Islam Rahat, Md Sohag Hossain, Mayen Uddin Mojumdar, Narayan Ranjan Chakraborty

**Affiliations:** Multidisciplinary Action Research Laboratory, Department of Computer Science and Engineering, Daffodil International University, Birulia, Dhaka 1216, Bangladesh

**Keywords:** Botanical taxonomy, Computer vision, Ecological monitoring, Floral diversity, Image recognition, Plant dataset, Species identification

## Abstract

We present the Tropical Flower Dataset: Seven Species from Bangladesh, a collection of 4,319 high-quality images containing seven tropical flower species: Rose (827), Bougainvillea (580), Marigold (717), Hibiscus (548), Crown of Thorn (583), Jungle Geranium (698), and Madagascar Periwinkle (366). All images were taken using a Redmi Note 11 smartphone at different locations within the Dhaka Division. The dataset is beneficial for flower classification and ecological purposes, encompassing diverse growth stages and distinct lighting conditions. Future work will focus on improving generalization through data augmentation and fine-tuning, aiming to enhance automated flower species recognition for ecological monitoring, biodiversity studies, and botany education.

Specifications Table

**Table:** 

Subject	Computer Sciences, Agriculture Sciences, Pharmaceutical Sciences.
Specific subject area	Computer Vision, Image Processing, Image Classification, Machine Learning.
Type of data	The images are in JPG format (total 4319 jpg files) having 3072 × 4080-pixel dimensions and 96 × 96 dpi image resolution.
Data collection	This dataset comprises a total of 4319 original flower images, which have been categorized into 7 different flower classes. The images were captured using Redmi Note 11 smartphone cameras.
Data source location	1. Savar, Dhaka Division, Bangladesh (Latitude: 23° 50′ 0.00″ N,Longitude: 90° 20′ 0.00″ E) 2. Hemayetpur, Savar, Dhaka Division, Bangladesh (Latitude: 23° 50′ 30.00″ N,Longitude: 90° 20′ 30.00″ E) 3. Ashulia, Savar, Dhaka Division, Bangladesh (Latitude: 23° 51′ 10.00″ N,Longitude: 90° 20′ 50.00″)*.*
Data accessibility	Repository name: Mendeley Data Data identification number: 10.17632/njfg9nh92t.1 Direct URL to data: https://data.mendeley.com/datasets/njfg9nh92t/1

## 1 Value of the Data

• Seven different tropical flower species are included in the dataset: rose, bougainvillea, marigold, hibiscus, crown of thorns, jungle geranium, and Madagascar periwinkle. This diversity offers a wealth of examples for classification tasks.
• This dataset contributes to studying the diversity of flowering plant species of tropical region in Bangladesh and opens up avenues for various types of future research. It features high-resolution images of seven species that take on various environmental conditions, ideal for background studies in ecology and florets in private gardens.
• When fitted with the appropriate algorithms, the images in the dataset can aid in the advancement of mobile applications for plant identification. These applications may assist farmers, botanists and conservationists by enabling rapid and accurate plant recognition in the field.
• Numerous feature extraction techniques are made possible by the high-resolution photos it holds.
• Plant taxonomy-related advances in computer vision and machine learning applications are made effective by this high-resolution photo's suitability for training complicated deep learning models.

## 2 Background

The motivation for compiling this tropical flower dataset stems from the need to document and analyse the diversity of flowering plant species in the regions of Savar, Hemayetpu**r**, and Ashulia, located within the Dhaka Division of Bangladesh. These areas, characterized by a mix of urban, suburban, and rural environments, present an opportunity to study plant species in diverse ecological settings. The data were generated through field surveys conducted to identify various flowering plants, including species such as Roses, Bougainvilleas, Marigolds, Hibiscuses, Crown of Thorns, Jungle Geraniums, and Madagascar Periwinkles. The study is based on a specific dataset, and while it demonstrates the accuracy of the algorithms used, further improvements could include adding more flower species, refining feature extraction methods, and enhancing the system's accuracy [1]. The challenges in flower recognition, arising from the diversity in species and visual similarities between flowers, were addressed by the researchers, as traditional classification methods struggled to handle these complexities. This work is a contribution to the emerging field of automated botanical image processing, providing a tool that may support ecological research [4] and studies in plant biodiversity an interactive flower recognition system is introduced, utilizing image processing and machine learning to classify flower species based on features such as color, shape, and texture. Users are enabled to upload images for instant identification, [2,3] which benefits education and botanical research. Traditional classification challenges are addressed, supporting ecological research and biodiversity monitoring, and advancing automated plant identification for conservation and environmental studies. [5] The research aims to improve the process of flower identification by using techniques from computer vision and machine learning. These techniques help extract key features from flower images, such as color, texture, and morphology, which are essential for differentiating between flower species [6] This dataset has theoretical background on ecological research and plant taxonomy focusing on species identification, environmental status and phenological records. The dataset was created methodologically using direct observations, GPS mapping and interviews with local inhabitants, leading to a highly detailed format of plants categorized into multiple key features such as growth type, colour of flower, flowering period and water requirement.

Introduced species plant data based on verifiable citizen observations can extend the value of prior efforts with local, mechanistic background information across its native range which will be adjunct to existing knowledge bases facilitating ecological studies, mobile plant identification applications, and educational use around plant science and environmental awareness in neighbouring jurisdictions beyond just biodiversity or conservation or horticulture topics segregated. In Table 1, a comprehensive understanding of the current work dataset, among other things. This can readily comprehend the significance of this dataset. Notably, the dataset contains seven tropical flower species including Crown of Thorns, Jungle Geranium, and Madagascar Periwinkle that are rare in other datasets. Image counts are also much higher for common species such as Rose (827 images) and Hibiscus (548 images) compared to other datasets, resulting in better diversity and robustness for machine learning applications. We compared our dataset to evaluate the effectiveness and uniqueness with related datasets from the works of I Gogul, VS Kumar,et al. (Rose, Marigold, Hibiscus) [2]. F Bozkurt,et al. (Rose) [11]. TH Hsu, CH Lee, et al. (Marigold, Hibiscus, Crown of Thorns, Madagascar Periwinkle) [5]. M. Hanafiah, M. A. Adnan, et al. (Rose) [10]. I Patel, S Patel, et al. (Hibiscus) [13]. Table 1 provides a detailed comparison of the datasets. Whereas most datasets focus on narrower species lists (like 3–4), our dataset covers a much wider array of species, making it suitable for classification approaches and ecological studies. Moreover, its high−resolution images (3072 × 4080 pixels) and tropical and subtropical flora geographic focus on Bangladesh provide further research utility. Additionally, the flexibility of the dataset to include natural and background-removed images further ensures that it can be used for both applications, in the wild and in a controlled experimental setting.

**Table 1:** Comparison with existing datasets.

SL	Flower	Our Dataset	I Gogul, VS Kumar, et al. [2]	F Bozkurt, et al. [11]	TH Hsu, CH Lee, et al. [5]	M. Hanafiah, M. A. Adnan, et al. [10]	I Patel, S Patel, et al. [13]
1	Rose	827	80	784	X	between 438 and 709 images	X
2	Bougainvillea	580	X	X	X	X	X
3	Marigold	717	80	X	10	X	X
4	Hibiscus	548	80	X	17	X	✔ (Not- Mentioned)
5	Crown of Thorns	583	X	X	9	X	X
6	Jungle Geranium	698	X	X	X	X	X
7	Madagascar Periwinkle	366	X	X	16	X	X

*Note:* (X) means data not available.

(✔) means data available but dataset number was not clearly mentioned.

## 3 Data Description

The Tropical Flower Dataset contains 4319 high-resolution images representing seven tropical flower species from Bangladesh: Roses, Bougainvilleas, Marigolds, Hibiscuses, Crown of Thorns, Jungle Geraniums, and Madagascar Periwinkles. These images were captured using a Redmi Note 11 smartphone across various natural environments within the Dhaka Division. Each image is labeled by species and formatted in JPG with a resolution of 3072 × 4080 pixels at 96 dpi, enabling detailed visual information for classification and ecological analysis. In Table3 The data collection process took place between October and November 2024, ensuring a variety of lighting and weather conditions, including sunny, weather. To capture a wide range of scenarios, images were taken at different times of the day: morning, noon, and afternoon. The dataset is systematically organized to include variations in background, lighting, and flowering stages, supporting robust training and testing for machine learning and computer vision applications. This diverse range of environmental factors makes the dataset suitable for the development of automated plant identification systems and offers a foundational resource for ecological monitoring, botanical research, and biodiversity studies. Table 1 provides a breakdown of the image count per species and comparison with others works, ensuring transparency in data distribution for potential model balancing and algorithmic fairness. The dataset's standardized format, high-quality images, and focus on common tropical flowers enable researchers and practitioners in fields such as agriculture, botany, and ecology to conduct experiments on flower classification, species recognition, and environmental adaptations. In Table 2, we have presented the visualization of other existing datasets. The final dataset, detailed in Tables 3 and 4, provides a visualization of the dataset's structure.

**Table 2:** Visualization of the other existing dataset.

**Table 3:** Visualization of the dataset. (With background).

**Table 4:** Visualization of the dataset. (Without background).

Following this consultation, we conducted a comprehensive data collection process, capturing images under varied natural lighting conditions. Data was collected from local botanical gardens, home gardens, and nurseries to ensure diversity and realism in the dataset. Each flower species was photographed from different angles and distances to account for variability in real-world scenarios. Table 5 contains the details of the data collection for each individual class of the flower.

**Table 5:** Data collection details.

Flower	Weather	Date	Time	Device	Location
Rose	Sunny	25–10–2024	Morning	Redmi Note 11	Ashulia (Latitude: 23° 51′ 10.00″ N,Longitude: 90° 20′ 50.00″)
Bougainvillea	Sunny	26–10–2024	Morning	Redmi Note 11	Ashulia (Latitude: 23° 51′ 10.00″ N,Longitude: 90° 20′ 50.00″)
Marigold	Sunny	28–10–2024	Noon	Redmi Note 11	Hemayetpur (Latitude: 23° 50′ 30.00″ N,Longitude: 90° 20′ 30.00″ E)
Hibiscus	Sunny	30–10–2024	Morning	Redmi Note 11	Savar (Latitude: 23° 50′ 0.00″ N,Longitude: 90° 20′ 0.00″ E)
Crown of Thorns	Sunny	01–11–2024	Afternoon	Redmi Note 11	Ashulia (Latitude: 23° 51′ 10.00″ N,Longitude: 90° 20′ 50.00″)
Jungle Geranium	Sunny	02–11–2024	Morning	Redmi Note 11	Savar (Latitude: 23° 50′ 0.00″ N,Longitude: 90° 20′ 0.00″ E)
Madagascar Periwinkle	Sunny	04–11–2024	Afternoon	Redmi Note 11	Hemayetpur (Latitude: 23° 50′ 30.00″ N,Longitude: 90° 20′ 30.00″ E)

## 4 Experimental Design, Materials and Methods

The data collection process for the Tropical Flower Dataset required several materials, which are mentioned in Table 5. This table includes the data collection locations, the weather conditions during the data collection, and the devices used throughout the process. The dataset comprises seven flower species: Rose, Bougainvillea, Marigold, Hibiscus, Crown of Thorns, Jungle Geranium, and Madagascar Periwinkle. To ensure the accuracy of flower identification and classification, our team consulted a botanist. During the consultation, we gathered critical insights into distinguishing features for each flower type, such as color, petal structure, and growth environment. Images were captured with minimal background clutter for easy background removal and postprocessing [7,8]. In addition, neutral, plain backgrounds were used at all times and the same lighting conditions secured equal or lesser shadows were present to improve performance of the segmentation. To accentuate flower isolation in the wild, flower length finder pointers and shallow depth-of-field were utilized through compositional adjustment of the camera position. These methods reduced contamination by nearby elements, such as leaves and stems*.*

## 5 Preprocessing

In order to perform background removal, the image preprocessing process begins by loading each image from an input folder and reading it into memory [14]. Backgrounds are removed using rembg library and the main subject is set with a transparent background. The image is then converted to RGBA and put on a plain white background to substitute any transparent areas (to make sure that all images look the same when it comes to model training). Now, in order to finish preprocessing, the image is converted to RGB (and thus dropping the alpha layer) and saved into the output folder. This approach normalizes every image to be ready for the subsequent steps in the ML pipeline including resizing and data augmentation, followed by model training [15]. To maintain consistency, the images of flowers in this dataset were captured using smartphone cameras at a same working distance from the object without zoom. During this data-gathering process, a total of 4319 flower images were collected. The background removal process utilized the rembg library, which uses deep learning-based segmentation to separate the flower from its background. Special care was taken to manually review and refine segmentation outputs to ensure that only the flower (and, in some cases, its associated leaves and stems, as relevant to species identification) remained in the foreground [9,10]. Inconsistent removal of leaves and stems was noted in the dataset and is acknowledged as a limitation. The pictures are all natural light photos where Capturing images under diverse weather conditions ensures representation of various lighting and external factors. Also, flowers from different areas of Savar, Hemayetpur and Ashulia were collected for representing various species in relation to diverse environmental conditions. Further investigations are necessary to explore the ecological roles and environmental adaptations of the species represented in this dataset.

## Limitations

One simple limitation of the dataset. It was the geographic bias first, it has been collected from specific sites at Savar, Hemayetpur and Ashulia so this may not represent the plant diversity of other parts of Bangladesh or any other ecosystems. Also, although smartphone cameras and image quality generally remained the same across studies, lighting conditions, weather behaviours, and background differences may have biased some images and reduced plant identification accuracy [11,12]. Data collection was also restricted to specific months, allowing for the exclusion of flowering plants in other seasons and potentially introducing an additional seasonal bias. Some species or groups of plants may also have been difficult to identify without expertise, particularly if the flower features used for identification were not fully visible because of environmental conditions or plant health. Data preprocessing is involved with the dataset so as to make it more useful for machine learning applications. Yet, such manipulation might restrict its practicality in real-world applications requiring plant identification systems to deal with natural backgrounds. Subsequent releases of the dataset will not only contain pre-processed images, but also the raw images itself, to mitigate this flaw and further increase applicability. Even though there aren't any photographs of damaged flowers in the collection yet, its expansion could make research on plant disease detection easier. By including both healthy and diseased specimens, early disease detection models might be developed, aiding in ecological and agricultural management.

## Ethics Statement

The authors declare that this research does not involve human subjects or animal experiments, nor the use of social media data. Data for this study are field-based and consisted of observations of flowering species in the wild. The authors also assure that the current work conforms to different ethical aspects for publication in Data in Brief. This research does not involve human participants as data were obtained from Twitter and all the animals involved in the study was acquired from one of the linked studies that have been previously published with proper ethical approval therefore, applicable informed consent, animal handling or social media data redistribution policies are irrelevant here.

## CRediT Author Statement

**Riazul Islam Rahat:** Conceptualization, Methodology, Software. **Md Sohag Hossain:** Data curation, Visualization, Writing. **Mayen Uddin Mojumdar:** Supervision, Methodology. **Narayan Ranjan Chakraborty:** Visualization, Investigation.

## Data Availability

Mendeley DataTropical Flower Dataset: Seven Species from Bangladesh for Classification and Ecological Research (Original data).
